# Commentary: Identification of Genes Linking Natural Killer Cells to Apoptosis in Acute Myocardial Infarction and Ischemic Stroke

**DOI:** 10.3389/fimmu.2022.944406

**Published:** 2022-07-07

**Authors:** Qi Li, Ting Huyan, Wei Zhang

**Affiliations:** ^1^ School of Life Sciences, Northwestern Polytechnical University, Xi’an, China; ^2^ Department of Anesthesiology and Perioperative Medicine, Henan Provincial People’s Hospital, People’s Hospital of Zhengzhou University, Zhengzhou, China

**Keywords:** natural killer cells, apoptosis, myocardial infarction, ischemic stroke, gene expression

Along with the widespread use of bulk and single-cell sequencing as well as microarray, massive data have been obtained with reasonable cost. How to extract the valuable information to answer specific scientific question is the concern by medical researchers. Numerous bioinformatics techniques have been developed at present to interpret the possible biological meaning of the enormous amount of data.

A recent research study by Feng et al. titled “Identification of Genes Linking Natural Killer Cells to Apoptosis in Acute Myocardial Infarction and Ischemic Stroke” identified the differentially expressed genes (DEGs) in acute myocardial infarction (AMI) and ischemic stroke (IS) by utilizing the public database and multiple bioinformatics analysis tools (1). They discovered that apoptosis-related genes such as *APAF1*, *IRAK3*, *ATM*, *CAPN1*, *IL1B*, *IL1R1*, *PRKACA*, *PRKACB*, and *TNFRSF1A* were negatively correlated with natural killer (NK) cells abundance in AMI and/or IS. There are many similarities between the two forms of ischemia disorders, making it worthwhile to compare them. This work established a complete bioinformatics analytic pipeline that facilitated the biomedical researchers to get disease-specific information.

However, it should be noted that the analyzed data are derived from peripheral blood samples, the majority of which are peripheral blood mononuclear cells (PBMCs). To what extent should the identification of immune cell proportion and DEGs in PBMC reflect the mechanism of AMI and IS? For example, when the IS occurs, the integrity of the blood–brain barrier (BBB) is compromised, and immune cells infiltrate into the ischemic brain tissue (2), part of them come from PBMCs. Consequently, the changes in peripheral blood may indirectly reflect the circumstances of the disease focus. In this article, the authors discovered that the proportion of infiltrated NK cells was lower in both AMI and IS than in the controls, and the abundance of infiltrated NK cells was significantly different between AMI and IS; therefore, it would be interested to know the following: 1. Is less NK cell infiltration the cause or result of the disease? 2. Do NK cells migrate easier through the BBB during IS? 3. What causes the difference in NK cell abundance between AMI and IS, which may represent the underlying differences in the pathogenesis of the two diseases?

NK cells are the most important innate immune cells that can directly recognize and kill the target cells regardless of presence of antibodies (3). It is also known that they stimulate the subsequent adaptive immune response by secreting cytokines (3). The dysfunction of NK cells may play a specific role in excessive inflammatory responses (4). It was shown that, in AMI, NK cells may play a role in mediating myocardial remodeling by regulating the inflammatory response (5), and NK cell is the latent reason of tissue damage in the focus of ischemia (6). There is an evidence showing that invariant NK T cells are pathogenic during AMI due to their pro-inflammatory NK cell receptor phenotype (7); therefore, we are very curious about the precise role of NK cells in AMI and IS, and whether dysfunctional NK cells contribute to the development of these two diseases. Consequently, it is crucial to identify the function of NK cells and secreted cytokine panel to reveal the mechanism of the secondary injury caused by inflammation in AMI and IS. It was known that NK cells could induce apoptosis in the target cell *via* the Fas/FasL, Tumor Necrosis Factor-α/Tumor Necrosis Factor Receptor (TNF-α/TNFR), as well as perforin/granzyme pathways (8). Therefore, NK-induced apoptosis is an essential aspect of its cytotoxicity. The following are unclear: Where the apoptosis-related genes mentioned in the article come from? Whose expression of specific apoptosis-related genes changes latent target cells, NK cells, or PBMCs? Changes of apoptosis-related genes in target cells and NK cells may present distinct biological meaning. For example, if the NK cell was the cell that expressed apoptosis genes, is NK cell apoptosis caused by ischemic injury and what role do apoptotic NK cells play at the site of the lesion? Given that the majority of samples selected in this article are PBMCs, we infer that the apoptosis-related genes are derived from PBMCs, and the correlation of PBMC apoptosis and ischemia injury needs to be further clarified.

Peripheral blood is the sample readily available with the minimal damage to human, and the gene markers that can reflect severity and prognosis of disease are relevant for scientists and clinicians. If the authors analyzed the DEGs to determine their ability to reflect the severity and prognosis of AMI and IS, then the results will be more meaningful.

Sequencing and bioinformatics analysis provides a great convenience for biomedical researchers, and the critical mechanism at the gene and protein level underlying diseases can be identified rapidly and affordably, which has immeasurably facilitated biomedical studies; however, wet laboratory experiments are still indispensable. The rigorous and repeatable experiments are the most essential to the advancement of biomedicine.

## Author Contributions

QL wrote the manuscript. TH provided important intellectual contribution. WZ revised the manuscript. All authors read and approved the submitted version.

## Funding

This work was funded by the Natural Science Basic Research Program of Shaanxi (grant number 2022JM-117); the Henan Province Middle-aged and Young Health Science and Technology Innovation Outstanding Youth Talent Training Project (grant number YXKC2021025); the China Postdoctoral Science Foundation (grant number 2020M673491) and the Fundamental Research Funds for the Central Universities (grant number D5000220170).

## Conflict of Interest

The authors declare that the research was conducted in the absence of any commercial or financial relationships that could be construed as a potential conflict of interest.

## Publisher’s Note

All claims expressed in this article are solely those of the authors and do not necessarily represent those of their affiliated organizations, or those of the publisher, the editors and the reviewers. Any product that may be evaluated in this article, or claim that may be made by its manufacturer, is not guaranteed or endorsed by the publisher.
